# Clinical features of human tuberculosis due to *Mycobacterium orygis* in Southern India

**DOI:** 10.1016/j.jctube.2023.100372

**Published:** 2023-04-15

**Authors:** Lydia Jennifer Sumanth, Christina Rachel Suresh, Manigandan Venkatesan, Abi Manesh, Marcel A. Behr, Vivek Kapur, Joy Sarojini Michael

**Affiliations:** a Associate Physician, Department of Clinical Microbiology, Christian Medical College, Vellore 632004, Tamil Nadu, India; b Consultant Microbiologist, Scudder Memorial Hospital, Ranipet, Tamil Nadu 632401, India; c Research Associate, Department of Clinical Microbiology, Christian Medical College, Vellore 632004, Tamil Nadu, India; d Associate Professor, Department of Infectious Diseases, Christian Medical College, Vellore, Tamil Nadu 632004, India; e Professor of Medicine, McGill International TB Centre, Montreal, Quebec H4A 3S5, Canada; f Professor, Infectious Diseases and Microbiology, The Pennsylvania State University, State College, PA 16802-3500, USA; g Professor, Department of Clinical Microbiology, Christian Medical College, Vellore, Tamil Nadu 632004, India

**Keywords:** *M. orygis*, South India

## Abstract

*Mycobacterium orygis* is a member of the *Mycobacterium tuberculosis* complex (MTBC) and causes tuberculosis in a variety of animals, including humans in South Asia. Here, we describe the clinical features associated with 8 human cases of whole genome sequence (WGS) confirmed *M. orygis* from a tertiary care hospital in South India during 2018–2019. The patient ages ranged from 9 to 51 years, with 5 females and 3 males included. All the patients had extrapulmonary disease with 2 having concomitant pulmonary involvement. Clinical improvement was documented after a full course of anti-tuberculosis therapy in 6 cases for whom follow-up was available. Taken together, the results show that *M. orygis* causes human tuberculosis in India, with a predominant extrapulmonary disease. Standardized molecular assays of this emerging member of the MTBC are needed to provide further information on the frequency of *M. orygis* infection in India and other countries where it is found in livestock and domestic wildlife.

## 1 Background:

Tuberculosis (TB) remains one of the leading causes of mortality from a single infectious agent and India leads the list of high-burden countries [1]. The *Mycobacterium tuberculosis* complex (MTBC) includes *M. tuberculosis sensu stricto* as well as a number of subspecies traditionally associated with non-human hosts such as *M. bovis* and the more recently named *M. orygis* [2]. In 1987, the first report of the ‘Oryx bacillus’ was described from a captive oryx in the Netherland zoo [3]. Subsequently, this organism and other genetically similar bacteria were named *M. orygis* in 2012 and recognized to be a distinct member of the MTBC [2]. *M. orygis* has been isolated from captive spotted deer, blue bull and from free ranging rhinoceros in Nepal [4], [5], from rhesus monkeys and cattle in Bangladesh [6], from cattle in South India [7]*,* spotted dear in Western India and Bison in Central India [8]. In contrast, human disease due to *M. orygis* has been mostly described on other continents. This includes 1 reported case of human to animal transmission from New Zealand [9], 8 cases of human tuberculosis in Australia [10], a human case of lymphadenitis due to *M.orygis* in USA [11], a retrospective report of 24 clinical isolates of *M. orygis* from the UK [12] and 5 cases in Norway [13]. Given that tuberculosis in migrants can present a different clinical spectrum due to the time after infection and the mode of detection [14], information on the clinical presentation in sites where the pathogen is endemic in livestock and wildlife is required to understand its impact in local populations.

Difficulty in differentiating *M. orygis* from *M. tuberculosis* and other members of the complex by traditional approaches has contributed to the paucity of information regarding the clinical presentation of *M. orygis*, especially in settings where sub-speciation of MTBC is not the norm [3], [12]. To fill this knowledge gap, this retrospective case series builds from the molecular epidemiologic investigations by Duffy *et al.* that identified 7 *M. orygis* amongst 940 MTBC samples screened using lineage-specific polymerase chain reaction (PCR) assays [15]. We describe a total of 8 human cases of TB due to *M. orygis* isolated from 1105 patients attending Christian Medical College Hospital, Vellore during a 10 -month period from October 1, 2018 through July 31, 2019.

Acid fast bacilli was identified by performing Ziehl Neelsen staining on the patient’s sample. Culture was performed by Mycobacterium growth indicator tube (MGIT) (BD-BACTEC MGIT 960 system), further MTB culture isolates were reconfirmed by Ziehl Neelsen staining and MPT64 TB antigen test (Abbott Bioline, Korea). For phenotypic drug susceptibility testing, cartridge based nucleic acid test (CB-NAAT) by Xpert MTB/RIF (Cepheid AB, Solna, Sweden) assay and MGIT method (BD-BACTEC MGIT 960 system) were employed. *M. orygis* was identified in each case by a multiplex real time PCR assay for a single nucleotide polymorphism (SNP) in *Rv0444c* (G698C) [15]. For whole genome sequencing, deoxyribonucleic acid (DNA) extraction was performed using QIAamp DNA Mini kit (QIAGEN, Hilden, Germany), subsequently, DNA was quantified with Qubit^TM^ 3 fluorometer (Thermo Fisher Scientific, CA, USA) using Qubit dsDNA HS kit (Invitrogen, Oregon, USA), and the DNA quality was verified by agarose gel electrophoresis. Preparation of Illumina sequencing libraries, barcoding of DNA library and sequencing were performed as described in Duffy *et al.* [15]*.* Polymorphisms associated with drug resistance mutations were characterized with TB profiler as described [16]. This case series provides the clinical context including descriptive epidemiology, clinical features, histopathology, radiological features and treatment outcomes of these 8 individuals presenting with TB caused by *M. orygis*.

## 2 Description of cases

**Fig. 1:**
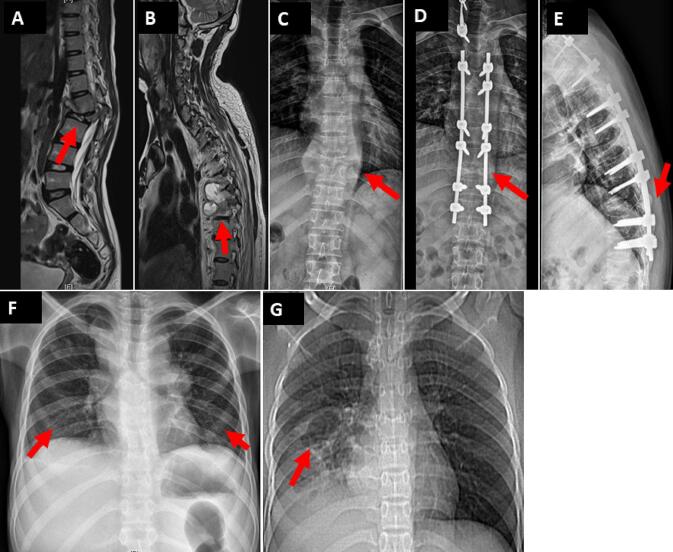
(**A**) Sagittal T2-weighted Magnetic resonance imaging (MRI) spine without contrast of 9-year-old female (case 1) showing near complete destruction of L2 (Lumbar) vertebra with pre- and paravertebral collection with bilateral psoas collection. (**B**) Sagittal T2-weighted MRI spine with contrast of 47-year-old male (case 2) showing interval increase in the destruction of T6-T7 (Thoracic) vertebral bodies, increase in extent of prevertebral, paravertebral, epidural and anterior subligamentous extension. (**C**) Thoracic spine anteroposterior (AP) view of 24-year-old male (case 4) showing paravertebral abscess at the level of T10 vertebra requiring decompression and stabilization. (**D**) Case 4 thoracic spine AP view post-surgical correction. (**E**) Case 4 thoracic spine lateral view post-surgical correction. **(F)** Chest radiography AP view of 16-year-old female (Case 6) shows bilateral lung nodular consolidation. **(G)** Computed Tomography (CT) of thorax without contrast of 16-year-old female (Case 8) shows consolidation with cystic changes of entire right lower lobe with branching nodular opacities in superior basal segment of right lower lobe.

*Case 1*: A 9-year-old female from West Bengal, presented with history of back pain for 6 months without neurological deficits. The patient had no reported history of contact with individuals with tuberculosis. On examination, the patient had gibbus deformity at L1-L2 (Lumbar) vertebrae, and magnetic resonance imaging (MRI) of spine showed near complete destruction of L2 vertebra with pre and paravertebral collection with bilateral psoas collection [Fig. 1(A)]. Smear from pus aspirated showed scanty acid-fast bacilli (AFB). Cartridge based nucleic acid test (CB-NAAT) showed low positive with rifampicin susceptibility and MTBC was identified by culture. The patient was treated with first-line anti-tubercular treatment [ATT; isoniazid (H), rifampicin (R), ethambutol (E) and pyrazinamide (Z)] for 9 months and recovered at follow up after 10 months.

*Case 2*: A 47-year-old male from Jharkhand presented with progressive difficulty in walking for 1 month and decreased sensation below the waist for 2 weeks. The patient also had difficulty passing urine and suffered from interscapular pain. The patient’s father was previously treated for TB of the spine. On evaluation the patient was found to have spondylodiscitis at T6-T7 (Thoracic) vertebral level with epidural collection [Fig. 1(B)]. Computed Tomography (CT) guided biopsy of the lesion was performed. Smear showed scanty AFB and CB-NAAT was low positive, rifampicin susceptible. MTBC was identified by culture and drug susceptibility test showed susceptibility to first line anti-tubercular antibiotics. Histopathology revealed necrotizing granulomatous inflammation. The patient was initiated on first line ATT plus levofloxacin for better bone penetration and was treated for 23 months. The patient also required surgical management of the thoracic cord compression. Symptomatic and radiological improvement was documented at 2 years follow up.

*Case 3*: A 21-year-old female from Jharkhand, presented with fever, dysuria, flank pain and increased frequency of micturition for 6 months. The patient reported no contact with individuals with TB. Ultrasonography of the abdomen showed multiple enlarged periportal and para-aortic nodes with multiple abscesses in the right kidney and hydroureteronephrosis suggestive of pyelonephritis. Smear from urine as well as lymph node biopsy tissue did not show any AFB. CB-NAAT from urine showed very low MTB, susceptible to rifampicin. Urine culture growth confirmed MTBC and drug susceptibility testing confirmed the isolate as susceptible to first line agents. Histopathology of lymph node showed necrotizing granulomatous inflammation. The patient was diagnosed as disseminated tuberculosis with involvement of the lymph nodes and genitourinary systems. Double-J ureteral stenting was performed, and the patient was initiated on first line ATT and was treated for 12 months. At follow up after 1 year the patient had resolution of bladder thickening, reduction in the size of renal abscesses, reduction in size of paraaortic and aortocaval lymph nodes, and symptomatic improvement.

*Case 4*: A 24-year-old male from Karnataka presented with fever, backache, loss of weight for 3 months and was diagnosed with paravertebral abscess [Fig. 1(C)]. The patient reported no contact with individuals with TB. CT guided biopsy of right paravertebral soft tissue at T10 vertebra was performed and smear did not show the presence of AFB. CB-NAAT was low positive, susceptible to rifampicin, and culture confirmed the growth of MTBC, susceptible to first line agents. Histopathology showed granulomatous inflammation. The patient was initiated on first line ATT, 20 days after onset of therapy patient showed paradoxical worsening of symptoms with multilevel spinal cord compression and impeding paraparesis. The patient underwent posterior decompression and stabilization [Fig. 1(D) and (E)] and continued ATT. The patient was treated for 1 year and reported good improvement on completion of therapy.

*Case 5*: A 47-year-old female from Bihar presented with fever, nausea and disseminated lymph node enlargement for 1 month. The patient reported no history of contact with individuals having TB. Smear of cervical lymph node showed no AFB and CB-NAAT was negative. Culture showed MTBC. Histopathology showed caseating granulomatous inflammation. The patient was initiated on first line ATT (renal adjusted dosage). However, the patient required continued management of chronic kidney disease due to interstitial nephritis. The patient was lost to follow up after the initiation of continuation phase 2 months after initial presentation.

*Case 6*: A 16-year-old female from West Bengal presented with dry cough and fever for 6 months. Chest radiography and CT scan of thorax and abdomen showed nodular consolidation of both lungs, generalized lymphadenopathy [Fig. 1(F)]. The patient reported no history of contact with individuals having TB. Smear from supraclavicular lymph node tissue was negative. CB-NAAT was very low positive, susceptible to rifampicin and the culture was positive for MTBC. Histopathology of lymph node showed caseating granulomatous inflammation. The patient was initiated on first line ATT. However, the patient was lost to follow up.

*Case 7*: A 51-year-old male from West Bengal presented with fever and cough for 3 months with loss of appetite and loss of weight with no history of contact with TB. The patient was diagnosed with disseminated tuberculosis involving lungs and abdomen. Ultrasonography of abdomen noted omental thickening, intraabdominal lymphadenopathy. Smear of sputum showed no AFB, CB-NAAT was low, susceptible to rifampicin and culture was positive for MTBC growth. The patient was started on ATT and at review after completing initiation phase the patient was advised to continue ATT for 1 year. After the completion of treatment, the patient showed significant symptomatic and radiological improvement.

*Case 8*: A 16-year-old female from Jharkhand presented with fever, cough and weight loss. The patient’s mother reportedly was diagnosed with TB 8 years prior. CT scan of thorax showed right lower lobe cystic changes and branching, nodular opacities with minimal right pleural effusion and hepatosplenomegaly [Fig. 1(G)]. Smear of sputum showed numerous (3+) AFB, CB-NAAT was high positive, susceptible to rifampicin and growth was confirmed on culture. Drug susceptibility testing (DST) was susceptible to first line agents. The patient was started on ATT and reviewed after induction phase. The patient was advised to continue ATT for 18 months. After completion of treatment, the patient improved, there were no new symptoms and the patient showed gain in weight.

## 3 Discussion

As shown in Table 1, of the 8 cases reported here, 5 were female and 3 were male. All were negative for HIV (Human Immunodeficiency Virus) infection. Risk factor of chronic native kidney disease was observed in 1 patient and no other risk factor was found in the other 7 cases. 1 patient was in the paediatric age group (9 years). Out of the 8 patients in this report, 7 were from the eastern part of the country and 3 reported history of household contact with TB. *M. orygis* has been thus far inferred to be a zoonotic disease, but history of consumption of unpasteurized animal products or exposure to infected livestock species, possible risk factors for acquiring zoonotic tuberculosis, was not elicited in any of the 8 cases.

**Table 1:** Description of demographic details, treatment, outcome, phenotypic and genotypic drug resistance in the patients.

Case no.	Age (yrs)	Sex	State name	Type of TB	Contact with active TB	AFB on Smear	SHRE DST	Treatment	Outcome	Genes associated with drug resistance mutations	SRA accession number
1	9	F	WB	E	None	Scanty	Susceptible	ATT completed	Improved	No mutation found	SRR10321149
2	47	M	JH	E	Father	Scanty	Susceptible	ATT completed	Improved	No mutation found	SRR10321130
3	21	F	JH	E	None	Negative	Susceptible	ATT completed	Improved	No mutation found	SRR10321134
4	24	M	KA	E	Uncle	Negative	Susceptible	ATT completed	Improved	gid115delC; 100%; Streptomycin	SRR10321143
5	47	F	BH	E	None	Negative	Not done	Initiation of continuation phase	Lost to follow up	No mutation found	SRR10321128
6	16	F	WB	E	None	Negative	Not done	Intensive phase	Lost to follow up	No mutation found	SRR10321152
7	51	M	WB	P and E	None	Negative	Not done	ATT completed	Improved	rrs-non coding transcript exon variant – streptomycin 51% frequency, rrs- non coding transcript exon variant- kanamycin, capreomycin, aminoglycosides, amikacin-45% frequency, rrs- non coding transcript exon variant -kanamycin, capreomycin, aminoglycosides, amikacin − 35% (rplC 4%, embB-0%)	SRR10321138
8	16	F	JH	P and E	Mother	Numerous	Susceptible	ATT completed	Improved	gyrB T500N; 41%; Quinolone	SRR21842101

Abbreviations: F, female; M, male; WB, West Bengal; JH, Jharkhand; KA, Karnataka; BH, Bihar; TB, tuberculosis; E, extrapulmonary; P, pulmonary; AFB, Acid Fast Bacilli; S, streptomycin; H, isoniazid; R, rifampicin; E, ethambutol; DST, drug susceptibility testing; ATT, anti-tubercular treatment; SRA, sequence read archive.

Direct smear for acid fast bacilli was negative in 5 of 8 cases making it essential to follow up with culture and/or molecular methods. CB-NAAT was positive in 7 cases with all susceptible to rifampicin. Though first line therapy maybe initiated based on the results of CB-NAAT, susceptibility for all drugs needs to be tested and appropriate changes in combination may be required. Whole genome sequencing did not identify signature polymorphisms in genes associated with resistance to anti-mycobacterial drugs in 5 isolates. A *gid* frameshift observed in case 4 with possible streptomycin resistance and the gyrB T500N was observed at 41% frequency in case 7 suggestive of potential for quinolone resistance, as discussed in Table 1. Phenotypic verification of this was however precluded by the failure to successfully revive the archived specimens. All 8 isolates had R463L and V469L katG alleles, suggesting that these represent the canonical *M. orygis* genotype but are not associated with drug resistance, given the phenotypic susceptibility of these isolates.

## 4 Concluding comments

To our knowledge, this is the largest reported series of *M. orygis* cases in humans from South Asia. *M. orygis* appears to be an important cause of Tuberculosis in a subset of the patients in India. Although the patients we describe were mostly from the Eastern part of the country, we cannot determine whether this is due to a bias in referral patterns to the hospital where they were managed. The patients with tuberculosis caused by *M. orygis* present primarily with extrapulmonary or disseminated forms of the disease. However, much remains to be understood about the disease spectrum and the molecular epidemiology of this infrequently reported MTBC lineage. Standardized molecular assays are needed to determine the prevalence of this emerging member of the MTBC and epidemiological factors promoting transmission needs to be elucidated.

## 5 Data summary

Genome sequences have been deposited at National Center for Biotechnology Information (NCBI) under BioProject database number PRJNA575883. The Sequence Read Archive (SRA) accession numbers are as described in Table 1.

## 6 Notes

**Financial support.** The current study was supported, in part, by the Bill & Melinda Gates Foundation (grant OPP1176950). MAB is supported by the Canadian Institutes for Health Research (grant FDN148362 ).

**Patient consent statement.** Waiver of the patient consent was sought for publication of the data collected. The study was approved by the institutional review board (IRB) of Christian Medical College, Vellore. (12695 dated 25.03.2020).

## Declaration of Competing Interest

The authors declare that they have no known competing financial interests or personal relationships that could have appeared to influence the work reported in this paper.
